# Therapeutic potential of PPARγ natural agonists in liver diseases

**DOI:** 10.1111/jcmm.15028

**Published:** 2020-02-07

**Authors:** Liwei Wu, Chuanyong Guo, Jianye Wu

**Affiliations:** ^1^ Department of Gastroenterology Putuo People’s Hospital Tongji University School of Medicine Shanghai China; ^2^ Department of Gastroenterology Shanghai Tenth People’s Hospital Tongji University School of Medicine Shanghai China

**Keywords:** liver diseases, natural agonists, PPARγ

## Abstract

Peroxisome proliferator‐activated receptor gamma (PPARγ) is a vital subtype of the PPAR family. The biological functions are complex and diverse. PPARγ plays a significant role in protecting the liver from inflammation, oxidation, fibrosis, fatty liver and tumours. Natural products are a promising pool for drug discovery, and enormous research effort has been invested in exploring the PPARγ‐activating potential of natural products. In this manuscript, we will review the research progress of PPARγ agonists from natural products in recent years and probe into the application potential and prospects of PPARγ natural agonists in the therapy of various liver diseases, including inflammation, hepatic fibrosis, non‐alcoholic fatty liver and liver cancer.

## 1 MOLECULAR STRUCTURE OF PEROXISOME PROLIFERATOR‐ACTIVATED RECEPTOR GAMMA (PPARγ)

The PPARs belongs to the superfamily of nuclear hormone receptors and is named for its activation, which is regulated by the peroxisome proliferators. There are three subtypes of PPARs (PPARα, PPARβ and PPARγ). These three subtypes of PPARs are expressed differently in different tissues. PPARα is mainly manifested in cardiomyocytes, hepatocytes, intestinal epithelial cells and renal tubule epithelial cells; PPARβ is found in many tissues, with the higher expression in the intestine, kidney and heart; and PPARγ is mainly expressed in adipose tissue.^1^

**Figure 1:**
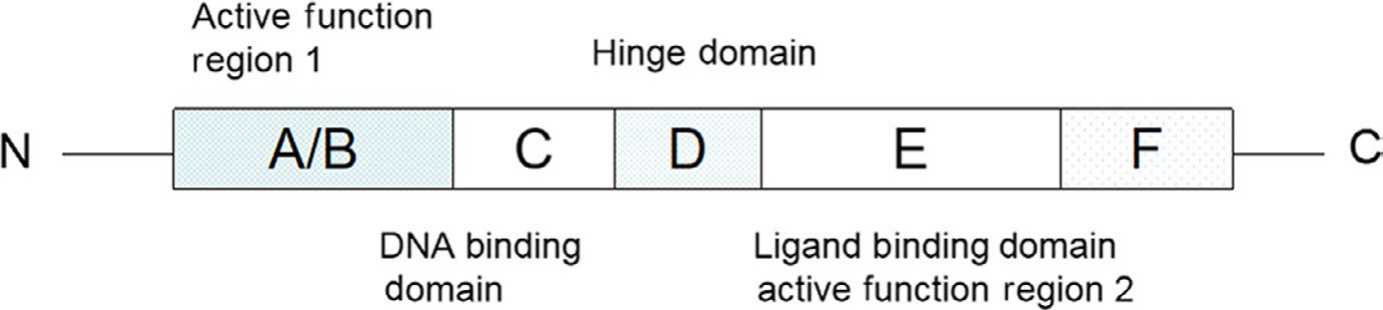
PPARγ structure. The A/B region, located at the N end of the receptor protein, is the active functional region. Region C is the DNA binding domain. Area D is the hinge domain. Region E/F, located at the end of C, is the ligand binding domain and contains a ligand‐dependent transcriptional activation functional region

PPARs always consist of four domains (A/B, C, D and E/F, Figure 1)The A/B region, located at the N end of the receptor protein, is the active functional region and differs among the subtypes and is independent of ligands. Region C is the DNA binding domain containing two zinc finger structures. Area D is the hinge domain. Region E/F, located at the end of C, is the ligand binding domain and contains a ligand‐dependent transcriptional activation functional region.^2^ The PPARγ gene can be transcribed into different PPARγ mRNAs and translated into two isoforms [PPAR γ1 and PPAR γ2].^3^

**Figure 2:**
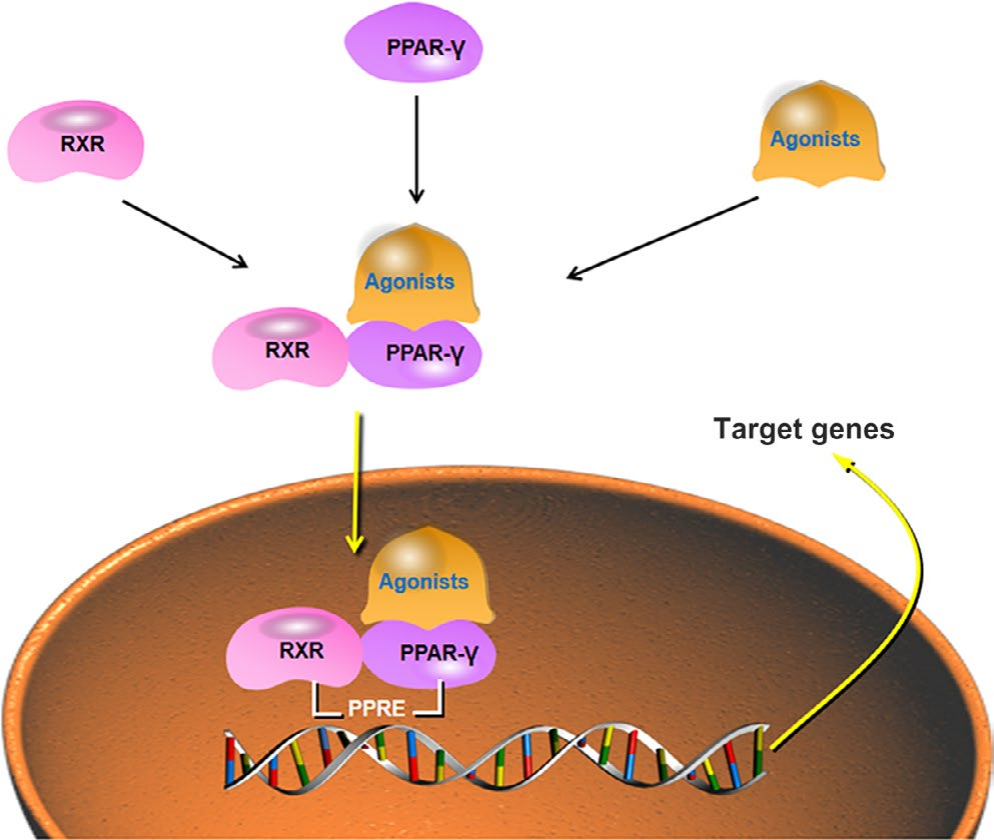
PPARγ activation. In normal cells, the PPARγ is located in the cytoplasm. After combining with its agonists and retinoid X receptor (RXR), the PPARγ complex translocates to the nucleus where it recognizes specific DNA sequence elements (peroxisome proliferator response element, PPRE) in promoters of target genes

After binding to ligands, PPARγ is activated and combines with retinoids X receptor (RXR) to form a heterodimer. Then, a series of synergistic factors are recruited and combined with the heterodimer to take part in regulating transduction. Typical endogenous ligands for PPARγ include prostaglandins, eicosanoids and fatty acids. At the same time, PPARγ can also directly activate specific genes or conduct gene transduction through DNA‐independent patterns (Figure 2).

## 2 FUNCTION AND CELLULAR ROLES OF PPARγ

The biological functions of PPARγ are complex and diverse, including regulation of lipid and carbohydrate metabolism, energy balance, inhibiting inflammation, inducing tumour cell differentiation and apoptosis, inhibiting tumour angiogenesis, anti‐fibrosis and anti‐atherosclerosis, reducing blood fat and blood pressure, improving heart failure and participating in ventricular remodelling. Thus, PPARγ is a current focus of research present. And indeed, there are a number of researchers, who have written review articles to shed more light on the power of PPARγ.

Semple reviewed the function of PPARγ and its variants in metabolic syndrome.^1^ In addition, Jia,^4^ Chigurupati^5^ and Vallée^6^ analysed therapeutic potential of PPARγ agonists in diabetes. PPARγ agonists improve insulin sensitivity and treat complications of diabetes. PPARγ can stimulate the differentiation of pre‐adipocytes into mature adipocytes and is closely related to adipogenesis in mature adipocytes.^7^ The beneficial role of PPARγ in regulation immunity was summarized by Samuel Philip Nobs,^8^ Chung,^9^ Abdelrahman,^10^ Giaginis^11^ and Staels.^12^ PPARγ inhibits pro‐inflammatory responses by macrophages, DCs, and T cells. Reka,^13^ Lecarpentier^14^ and Heudobler^15^ reviewed the implications for PPARγ in cancer therapy and prevention. Activation of PPARγ by agonists has the ability to inhibit cell proliferation and growth based on the ability to induce differentiation. A number of in vitro and in vivo experiments have shown that PPARγ is expressed in tumour cells and can inhibit the growth of cancer cells after activation, such as breast cancer,^16^, ^17^ pancreatic cancer,^18^ colon cancer^19^, ^20^ and gastric cancer.^21^ Additional results confirmed that decreased expression of PPARγ was found in activated hepatic stellate cells (HSCs), suggesting that the increased expression and activity of PPARγ promoted the recovery of activated HSCs to a resting state.^22^, ^23^, ^24^, ^25^ Among the multiple biological responses involved, PPARγ plays a corresponding role by regulating the expression of signalling pathways, including JAK‐STAT, NF‐kB, nuclear factor of activated T cell, AP‐1, PI3K, leptin and adiponectin. Therefore, PPARγ is of vital importance when making a diagnosis and selecting treatment for related diseases.

## 3 PPARγ AGONISTS FROM NATURAL PRODUCTS

Because of the significant role of PPARγ in diseases, the identification of PPARγ agonists is regarded as targets of numerous drug development works. Large amounts of fatty acids and fatty acid derivatives can activate PPARγ. Among the PPARγ activators, long‐chain polyunsaturated fatty acids always show better effects, such as eicosanoids [8‐S‐hydroxyeicosatetraenoic acid and leukotriene B4]. Also, PPARγ can be activated by several prostanoids, such as 15‐deoxy‐Δ12, 14‐prostaglandin J2 (15d‐PGJ2) and 15‐hydroxyeicosatetraenoic acid. The effect of 15d‐PGJ2 has been widely recognized.^11^ Thiazolidinediones (TZDs) are synthetic ligands of PPARγ and are well‐known for excellent potency in regulating blood glucose levels and insulin sensitivity.^26^ However, the undesirable side effects, such as fluid retention, weight gain, cardiac hypertrophy and hepatotoxicity, have limited the clinical use of TZDs.^5^ Thus, searching for drugs with a similar clinical function, but fewer side effects has become a new direction of effort. Natural products are rich sources of drug discovery; thus, natural products are a focus of research.^27^, ^28^

Previous studies have successfully demonstrated various PPARγ agonists from natural resources by using reporter gene assays, pharmacophore models, silicon screening and virtual screening approaches. A cell‐based luciferase reporter system may become a suitable method to detect bioavailability of nuclear receptors, including PPARs.^29^ Rasmus^30^ demonstrated that the pharmacophore model can be used to select novel PPARs agonists. In addition, Jang and Peng^31^, ^32^ identified promising PPARγ agonists on the basis of structure analyses. Since the first time that virtual screening (VS) was used to identify novel PPARγ agonists by Salam et al,^33^ more and more researchers have begun using in silico methods alone or combined with other approaches, such as in vivo or in vitro experiments,^34^, ^35^ structure analyses^36^, ^37^ and some databases,^36^ to find novel agonists as potential candidates to treat diseases.^38^ The functionality of some approaches has been verified.

To review PPARγ agonists from natural products we checked the database, DrugBank (http://www.drugbank.ca), which combines bio‐ and chem‐informatics. Table 1 shows our results. Resveratrol, curcumin, isoflavone, cannabidiol, nabiximols and medical cannabis have been confirmed to have the agonist role.

**Table 1:** PPARγ natural agonists in DrugBank

DrugBank ID	Name	Structure	Function description	References
DB02709	Resveratrol	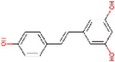	Antioxidant, anti‐inflammatory, anti‐cancer	42, 95, 112, 113, 114, 115
DB11672,14635	Curcumin, Curcumin sulphate	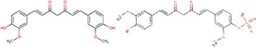	Anti‐inflammatory, antioxidant, metabolic regulation	68, 72, 73, 116, 117, 118, 119, 120
DB12007	Isoflavone	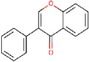	Anti‐inflammatory, antioxidant, anti‐cancer	108, 121, 122
DB14009(09061,14011)	Medical Cannabis (Cannabidiol, Nabiximols)	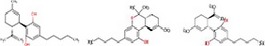	Antioxidant, anti‐inflammatory, neuroprotective	122, 123, 124, 125, 126, 127, 128, 129, 130, 131, 132, 133, 134

Not surprisingly, an abundance of research efforts has been undertaken to explore the potential applications of full or partial PPARγ natural agonists. Table 2 exhibits the natural agonists and their functions, which have been discussed in recent years. After reviewing the reported agonists, we found that the majority are flavonoids or isoflavonoids. Most of the other agonists are stilbenes, polyacetylenes, amorfrutins, sesquiterpene lactones and derivatives of diterpenequinone. The diversity of agonists depends on the large size of the LBD binding pocket and its flexibility.

**Table 2:** Discussed natural agonists of PPARγ (from 2010 to 2019)

Functions	Agonists	Years	References
Anti‐cancer	*Chromolaena odorata*, Luteolin, Stereoisomers ginsenosides	2012	136, 137, 138, 139, 140
	*Turbinaria ornata* and *Padina pavonica*	2015	141
	Resveratrol	2016, 2019	42, 95, 113, 115
Anti‐fibrosis	Puerarin	2013, 2017	74
	Piperine	2017	143
	Berberine	2018	144
Anti‐inflammation	Monascin	2011, 2014	145, 146, 147
	Astaxanthin, Ankaflavin, Biochanin A, Cullin‐3, Danhong, Daidzein	2012	148, 149, 150, 151, 152, 153
	Ursolic acid, Epigallocatechin gallate, Monascin	2013	154, 155, 156
	Rhizoma Dioscoreae Nipponicae polysaccharides, Harpagoside, Tectorigenin, Chrysin	2015	157, 158, 159, 160
	Huangkui, Tripchlorolide, *Kochia scoparia* and *Rosa multiflora*, Resveratrol, Chrysin, Daidzein	2016	115, 121, 161, 162, 163, 164
	Astragalus, Fraglide‐1, Madecassic acid, Epigallocatechin Gallate, Hesperetin	2017	66
	Isoprenylated flavonoid, chrysogenum J08NF‐4, *Portulaca oleracea* L., Betulin, *Terminalia arjuna*, Naringin	2018	69, 169, 170, 171, 172, 173
	Beta‐caryophyllene, Wogonin, Resveratrol, Hesperetin	2019	113, 174, 175, 176
Metabolism regulation	Cerco‐A, Mycophenolic acid, Fructus Schisandrae, Monascin	2011	156, 177, 178, 179
	Ankaflavin, Astaxanthin, Danhong	2012	149, 150, 152
	Amorfrutin, Honokiol, Monascin	2013	156, 180, 181
	Chebulagic acid, Monascin	2014	145, 147, 182
	Kaempferol, *Lonicera japonica* Thunb, Quercetin, Tectorigenin	2015	160, 183, 184
	Osthole, Isorhamnetin, Huangkui, Saponins and sapogenins, Resveratrol, quercetin	2016	95, 162, 185, 186, 187, 188
	ZINC13408172, 4292805, 44179 and 901461, Lycium, Astragalus, Tetrahydrocannabinolic acid, Astragaloside IV	2017	168, 189, 190, 191, 192
	Betulin, Chlorogenic acid, Isoprenylated flavonoid, Gentiopicroside, Geranylgeraniol, Moringa concanensis Nimmo, *Terminalia arjuna*, Saponins and sapogenins	2018	169, 170, 172, 193, 194, 195, 196
	*Kaempferia parviflora*, Moringa concanensis Nimmo, Resveratrol	2019	42, 95, 197, 198

Meanwhile, there are new trends in the treatment of liver disease which are using dual PPARα/γ or PPAR δ/γ agonists and pan agonists to enhance treatment efficacy.^39^, ^40^ Of note, synthetic dual or pan PPAR agonists were discontinued due to adverse events.^41^ It has been showed that resveratrol,^42^ carvacrol,^43^ osthole,^44^ dark tea extracts,^45^ isoprenols,^46^ pseudolaric acid B,^47^ mulberry leaf water extract, Korean red ginseng, banaba leaf water extract,^48^ and cannabinoids^49^ activate two or three isotypes of PPARs, and can therefore be used for regulate metabolism. And the compound functions are discussed below.

The liver is the centre of bio‐transformation and detoxification of numerous metabolites and toxicants. Exposure to high levels of exogenous or endogenous toxins may lead to liver damage, which ranges from a transient elevation of liver enzymes to hepatic inflammation, fibrosis, cirrhosis and cancer. Although the expression of PPARγ is always at a low level in liver, PPARγ agonists exhibit various PPARγ‐dependent or PPARγ‐independent effects in liver.^50^ In addition, researches on our team have focused on the prevention and therapy of liver diseases in recent years. We also have published some reports on the effects of PPARs in liver diseases. The protective effects of many Chinese herbal medicines, such as quercetin,^51^, ^52^, ^53^ oleanolic acid,^54^ proanthocyanidin B2,^55^ epigallocatechin‐3‐gallate,^56^ isorhamnetin^57^ and genistein,^58^ in liver diseases have been confirmed by our studies.

In fact, some of the Chinese herbal medicines or plants extracts have been reported to have a close relationship with PPARs, and a range of PPARγ activating natural products were recently recognized that possess a great potential to be further explored for the therapeutic effectiveness in liver diseases; but it has not thoroughly reviewed, and its natural agonists have been evaluated even less. Even, few reviews of the effects of PPARγ natural agonists in liver disease have been published. Understanding the role natural products play, as well as their therapeutic potential for fighting liver diseases, including hepatitis, fibrosis, fatty liver and liver cancer, is critical for future progress. Therefore, our present review summarizes the latest research progress of PPARγ agonists from natural products in recent years and explores the application prospect of PPARγ natural agonists in the treatment of liver diseases.

## 4 PPARγ NATURAL AGONISTS AND LIVER DISEASES

### 4.1 PPARγ natural agonists in hepatitis‐associated inflammation

**Figure 3:**
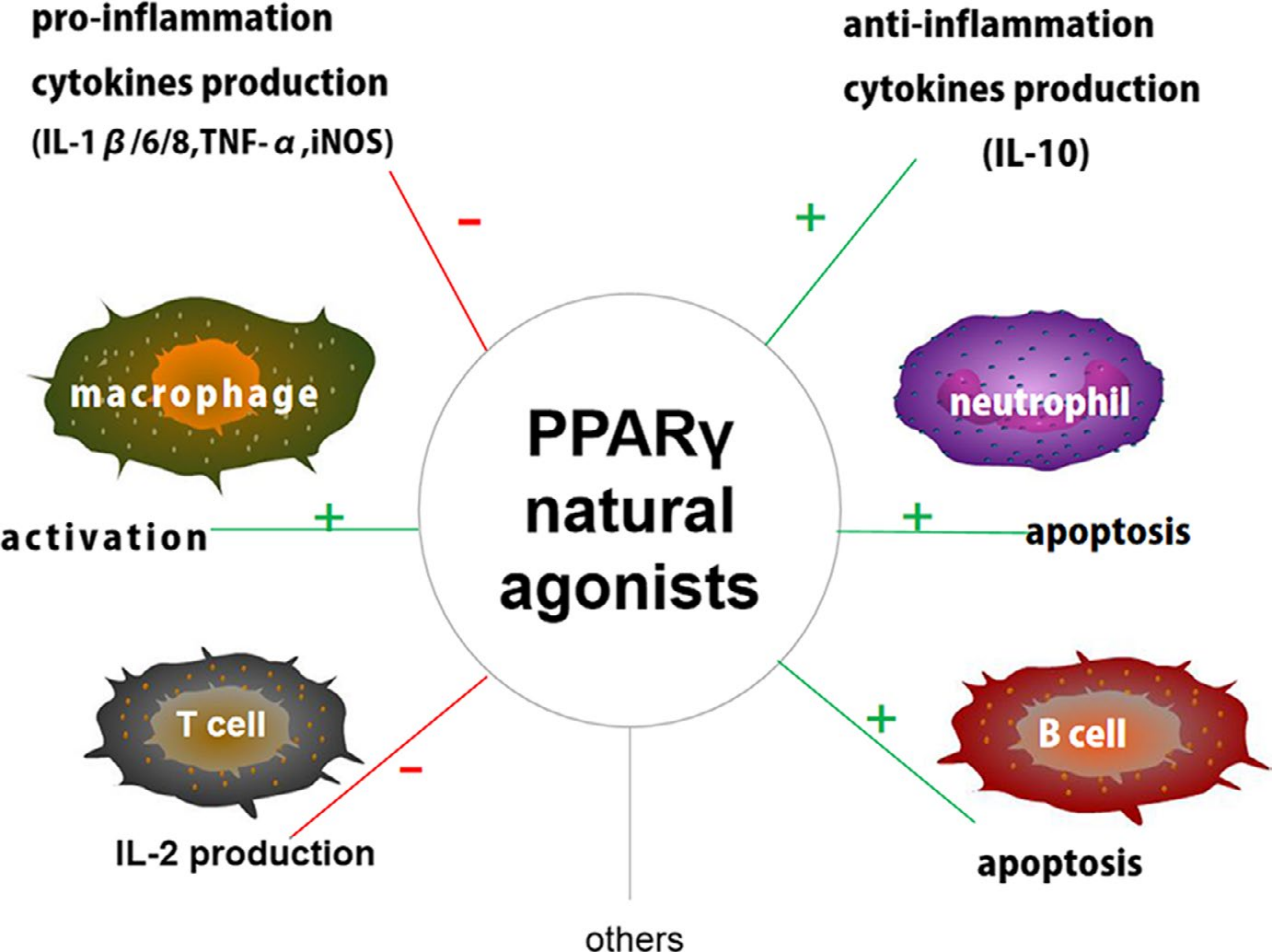
PPARγ natural agonists and inflammation. PPARγ natural agonists can regulate inflammatory responses. PPARγ natural agonists promote the activation of macrophages, the apoptosis of neutrophils and B cells, and the expression of some anti‐inflammatory cytokines. In addition, PPARγ suppresses the function of T cells and decreases the expression of some pro‐inflammatory cytokines

Inflammation is provoked by pathogenic agents, physical or chemical harm, and ischaemic or autoimmune injury, and it is a vital response for protection. The role of PPARγ in the regulation of inflammatory responses has received particular attention. PPARγ appears to be expressed in many cell types of the immune system, such as macrophages, dendritic cells, platelets, T cells and B cells.^59^ In addition, PPARγ has been shown in numerous studies to affect the expression of pro‐inflammatory, anti‐inflammatory and pro‐resolving cytokines.^60^, ^61^, ^62^, ^63^ (Figure 3).

Feng reported that apigenin activates PPARγ and ameliorates inflammation via regulation of macrophage polarization.^64^ Apigenin (4,5,7‐trihydroxyflavone) is a plant flavonoid abundant in fruits and vegetables that acts as a PPARγ modulator by binding and activating the PPARγ. Moreover, PPARγ is regarded as a modulator of macrophage polarization. Apigenin activates PPARγ and inhibits p65 translocation into the nucleus, favouring M2 macrophage polarization. The ability of apigenin in reversing M1 macrophages into M2 macrophages was confirmed based on in vivo experiments in mice.^65^ Apigenin decreased the secretion levels of interleukin(IL)‐1β, IL‐6, IL‐12 and TNF‐α both in vitro and in vivo. Hesperidin is a flavanone glycoside in citrus fruits. When detecting the effect of hesperidin in diethylnitrosamine‐induced hepatocarcinogenesis, Mahmoud found that hesperidin ameliorates oxidative stress and inflammation, dramatically up‐regulates the expression of PPARγ, and significantly prevents liver damage.^66^, ^67^ The anti‐inflammatory effect of curcumin, a natural polyphenolic compound, was reported by El‐Naggar et al.^68^ In streptozotocin‐induced diabetic rats, the up‐regulation of alanine aminotransferase, aspartate aminotransferase, cyclooxygenase, transforming growth factor‐β1 and nuclear factor kappa B were reversed by curcumin via its promotion of PPARγ expression. In an investigation of the jellyfish‐derived fungus, *Penicillium chrysogenum* J08NF‐4, researchers described a new meroterpene derivative, chrysogenester, which has been defined as a PPARγ agonist. In this study, Lius found that chrysogenester activates PPARγ in Ac2F liver cells and increases nuclear PPARγ protein in RAW 264.7 macrophages. Chrysogenester inhibits phosphorylation of the NF‐κB and suppressed the expression of pro‐inflammatory cytokines, including NO, TNF‐α, IL‐1β and IL‐6.^69^

These reports confirmed the function of PPARγ natural agonists in liver inflammation. The anti‐inflammatory properties of betulin, biochanin A, epigallocatechin gallate, harpagoside, madecassic acid, monascin, resveratrol, rhizoma dioscoreae nipponicae polysaccharides and ursolic acid, which can increase the expression of PPARγ, have been explored by many other scientists. These findings provide evidence for the application prospect of PPARγ natural agonists in inflammatory liver diseases.

### 4.2 PPARγ natural agonists in liver fibrosis

**Figure 4:**
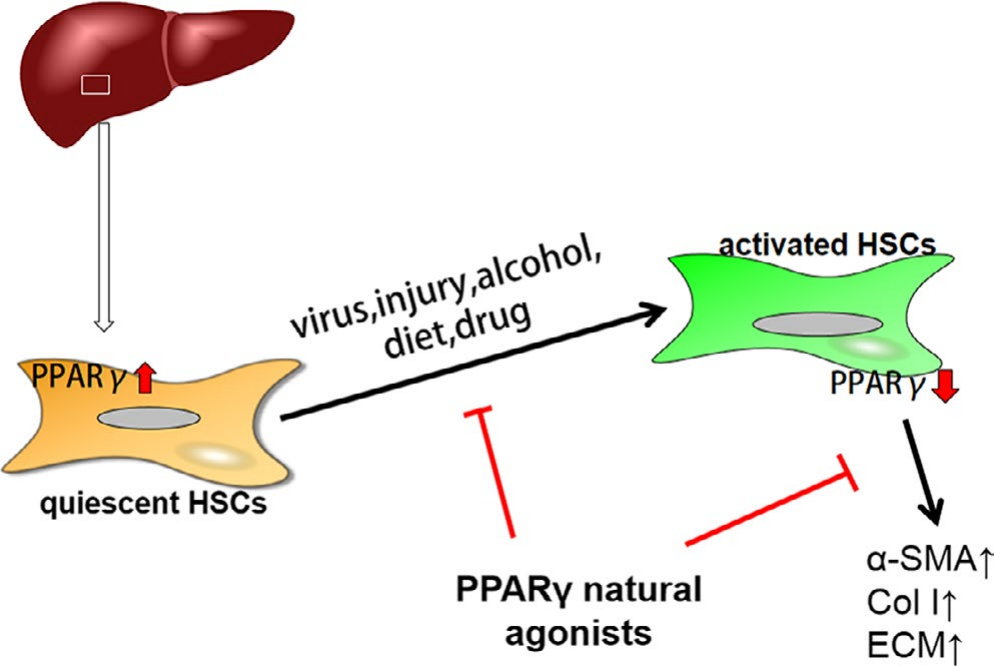
PPARγ natural agonists and liver fibrosis. Activation of HSCs is closely connected with viral infections, injury alcohol, diet and drugs. PPARγ is highly expressed in quiescent HSCs; however, PPARγ is suppressed during the process of fibrosis. PPARγ natural agonists block HSC activation and reduce collagen deposition during hepatic fibrogenesis

Liver fibrosis is a chronic and dynamic pathophysiological process, and commonly, excessive secretion and deposition of matrix proteins by HSCs is a pivotal step. Liver fibrosis is closely connected with hepatitis virus infection, alcohol and lipids. The expression of PPARγ is high expression in quiescent HSCs; however, PPARγ is suppressed during fibrosis process. Studies have shown that PPARγ activation blocks HSCs activation and reduces collagen deposition during hepatic fibrogenesis. Thus, PPARγ is an effective target in anti‐fibrosis therapy.^70^ Also, most PPARγ agonists from nature are partial agonists and always play a biological role by regulating the expression of a variety of genes, resulting in achieving better results. Thus, more and more authorities believe PPARγ agonists could become available therapeutic agents (Figure 4).

Curcumin, for acid polyphenols, is a yellow pigment in turmeric. Zheng and Chen have verified curcumin function inducing PPARγ expression in activated HSCs and suppressing extracellular matrix production (ECM). They found that curcumin could stimulate the trans‐activation activity of PPARγ, and thus reduce HSC proliferation, induce apoptosis, down‐regulate the expression of ECM gene expression and regulate pathways of TGF‐β and connective tissue growth factor.^71^, ^72^ Guo et al described the anti‐fibrotic role of puerarin, an active ingredient from kudzu root. Puerarin effectively attenuated liver damage by up‐regulating PPARγ expression in CCl_4_‐induced hepatic fibrosis. Puerarin can reverse the changes in serum hepatic enzyme activity, reduce ECM deposition and regulate the expression of matrix metalloproteinases (MMPs) and tissue inhibitors of metalloproteinase (TIMPs).^73^ Monascin is derived from monascus‐fermented secondary metabolites. It has been shown that monascin rescues the inhibited expression of PPARγ. In HSCs from carboxymethyllysine‐induced fibrosis, monascin attenuates α‐smooth muscle actin and reactive oxygen species generation. Monascin may slow or even block the progression of liver fibrosis through activation of PPARγ.^74^ Chois reported that capsaicininhibits liver fibrosis by restraining the TGF‐β1 pathway expression via activation of PPARγ. This report described the protective effect of capsaicin. The mechanism of action of capsaicin includes the reduction of oxidative stress and inflammatory response, induction of HSCs apoptosis and repression of ECM production.^75^

### 4.3 PPARγ natural agonists in liver cancer

More than one decade ago, PPARγ was reported to be closely related to the formation of liver tumours in animals. Researchers found that oestrogen can activate PPARγ by inducing the formation of the metabolite of prostaglandin D2, then activated PPARγ can promote the proliferation of peroxidase bodies, finally causing oxidative DNA damage. This process is closely related to the formation of hepatic tumours.^76^ However, with the deepening of research, people have different definitions about the role of PPARγ in the development of liver cancer.^77^, ^78^ Koga^79^ reported that the expression of PPARγ in liver cancer was very similar to that in surrounding non‐tumorous cirrhotic liver; however, the number of cases was small. Schaefer^80^ and Lin^81^ found that PPARγ is highly expressed in hepatic cancer tissues and in HCC cell lines, and the inhibition of PPARγ function could cause HCC cell death. At the same time, other papers analysed the expression of PPARγ in human HCC tissues and adjacent non‐tumorous liver tissue, and found a significant decrease in HCC tissues, thus showing us that PPARγ ligands, including thiazolidinediones. TZDs and 15‐deoxy‐Δ12,14‐prostaglandin J2 inhibit growth and induce apoptosis of liver cancer cells.^82^, ^83^, ^84^, ^85^, ^86^, ^87^

When scientists shifted their perspective to natural agonists of PPARγ, the potential in HCC therapy was shown. Avicularin is a bioactive flavonoid from various plants. Researchers use Huh7 cells to investigate the effect of avicularin in HCC. The results indicated that avicularin treatment decreased cell proliferation, inhibited cell migration and invasion in HCC and induced cell apoptosis via inhibiting the G0/G1‐phase cells and decreasing the accumulation of S‐phase cells. Moreover, the demonstrated anti‐cancer efficacy of avicularin was at least partly dependent on its activation of PPARγ activities.^88^ Another flavonoid, hispidulin, exhibits potent cytotoxicity towards a variety of human cancers. Hans confirmed the protective effect of hispidulin on HCC both in vitro and in vivo. Hispidulin triggered apoptosis, inhibited cell migration and invasion, and activated PPARγ signalling. The animal experiments showed that hispidulin administration could suppress tumour growth and lung metastasis.^89^ Huangs studied the combined effects of chrysin and apigenin, both of which are found in *Morinda citrifolia*, in liver cancer. These two drugs were used in both in vivo and in vitro experiments, and authors found they could inhibit cancer cell growth, disorganize cell cycle distributions and suppress cancer cell migration. The combined effects were better, compared with either alone.^90^ Vara team detected the anti‐proliferative effects of cannabinoids in hepatocellular carcinoma on HepG2 and HUH‐7 cell lines in vitro and in vivo. Δ9‐tetrahydrocannabinol and JWH‐015 are two famous cannabinoids, and they could inhibit cancer cell proliferation and induce autophagy. The activity and intracellular level of PPARγ were increased by them, and the effects can be abolished by a PPARγ inhibitor.^91^

The studies on the favourable effects of PPARγ natural agonists for HCC were few, and researches for several other types of cancer are listed in Table 3.^16^, ^92^, ^93^, ^94^ To some extent, they can also demonstrate the potential of PPARγ natural agonists as anti‐liver cancer agents.

**Table 3:** Anti‐cancer effect of PPARγ natural agonists

Agonists	Cancer type	Function	References
Cladosporol A	Colorectal cancer cell	Inhibit proliferation, up‐regulate 3 p21waf1/cip1 gene expression, inactivate β‐catenin/TCF pathway	94
Morusin	Breast cancer cell	Inhibit proliferation, induce adipogenic differentiation, apoptosis and lipoapoptosis of cancer cells, up‐regulate expressions of C/EBPβ	16
Carotenoids	Leukaemia K562 cells	Inhibit proliferation, decrease the viability, induced G0/G1 cell cycle arrest, up‐regulate the expression of Nrf2	93
Chrysin	Breast cancer cell	Inhibit proliferation, decrease the viability, inhibit epithelial‐mesenchymal transition	91
6‐Shogaol	Breast and colon cancer cell	Inhibit proliferation, induced G2/M cell cycle arrest	19
Bitter gourd seed	Colon cancer cell	Inhibit proliferation, induce apoptosis and up‐regulate GADD45, p53	20
Deoxyelephantopin	Hela cells	Inhibit proliferation, induce apoptosis and cell cycle arrest at G(2)/M phase	199
Dihydroartemisinin	Colon cancer cell	Inhibit proliferation, induce apoptosis	200
isoprenols	Colon cancer cell	Induce apoptosis	201
Hydroxysafflor‐Yellow A	Gastric carcinoma cell	Inhibit proliferation, induce apoptosis and cell cycle arrest at G0/G1 phase	202
Luteolin	Colorectal cancer cell	Luteolin‐mediated OCTN2 expression and activity potentiate the sensitivity of cancer cells to oxaliplatin	203
Lycopene	Prostate cancer cell	Inhibit proliferation	204

### 4.4 PPARγ natural agonists in non‐alcoholic fatty liver disease (NAFLD)

Fatty liver disease, due to input/output imbalance of hepatic free fatty acid(FFA) metabolism, is regarded as one of the most common chronic liver diseases worldwide. Insulin resistance and oxygen stress are regarded as the central to development. The multi‐layer and multi‐angle function of PPARγ have been confirmed by many researchers.^95^, ^96^ As we mentioned above, PPARγ activation down‐regulate inflammatory response,^97^ inhibit HSCs activation,^98^ increase energy expenditure^99^ and increase insulin sensitivity.^100^ PPARγ activation could stimulate fatty acid oxidation in the liver.^101^, ^102^ These are positive roles of PPARγ. At the same time, in vivo experiments for deletion or overexpression of PPARγ exhibited its prosteatotic role in the development of NAFLD or NASH.^103^, ^104^, ^105^ PPARγ also regulates lipid deposition in liver and other tissues.^106^ Utilizing the positive effects of PPARγ while limiting its negative effects by targeting other PPARs has paved the way for the development of a new batch of dual and pan agonists. Some researchers have set their sights to natural dual and pan PPAR agonists. There are some cell studies showing that soy isoflavones exhibit antidiabetic and hypolipidemic effects by activating both PPARα and PPARγ.^107^ Guozhu^42^ reported that resveratrol suppresses oleate‐induced total cholesterol accumulation in macrophages by activating PPARα/γ signalling pathway, and it was confirmed that resveratrol could prevent hepatic steatosis in NAFLD.^108^ Polyphenolic compounds from different sources showed apparent effects on PPAR expression and affect lipid accumulation in high‐fat‐fed mice.^109^ Bavachinin is a natural pan PPAR agonist that increase effectiveness of TZDs or fibrates when regulating carbohydrate and lipid metabolism in diet‐induced obese mice.^110^

## 5 CONCLUSION

Natural products have been and continue to be rich sources for drug discovery. Natural agonists of PPARγ have confirmed anti‐inflammatory, antioxidant properties, anti‐fibrosis, anti‐tumour and metabolism regulation effects. These beneficial effects may be partly due to the role of PPARγ in pathophysiological processes. Both experimental and clinical research results have indicated PPARγ agonists from natural products play vital roles in their protective effects in liver diseases. Besides, dual PPARα/γ or PPAR δ/γ agonists and pan agonists have draw researchers’ attention, and sometimes they have better curative effects.

However, the limitation of this review is that there are few studies on the treatment of liver diseases with PPARγ natural agonists. Because PPARγ and the target genes of natural products are diverse, it is likely that many other mechanisms contribute to their beneficial effects in these and other disease models. A lot of ongoing research efforts are trying to broaden our horizons to better understand the role of PPARγ systematically.

## CONFLICT OF INTERESTS

The authors report no conflicts of interest in the present study.

## AUTHOR CONTRIBUTION

Liwei Wu wrote the manuscript and made the original tables and figures. Chuanyong Guo and Jianye Wu revised the manuscript and tables and figures. All authors read and approved the final manuscript.

## Data Availability

All data included in this study are available.

## References

[1] Semple RK , Chatterjee VK , O'Rahilly S . PPAR gamma and human metabolic disease. J Clin Invest. 2006;116:581‐589. 16511590 10.1172/JCI28003 PMC1386124

[2] Hallenborg P , Petersen RK , Kouskoumvekaki I , Newman JW , Madsen L , Kristiansen K . The elusive endogenous adipogenic PPARgamma agonists: lining up the suspects. Prog Lipid Res. 2016;61:149‐162. 26703188 10.1016/j.plipres.2015.11.002

[3] Goto T , Kim YI , Takahashi N , Kawada T . Natural compounds regulate energy metabolism by the modulating the activity of lipid‐sensing nuclear receptors. Mol Nutr Food Res. 2013;57:20‐33. 23180608 10.1002/mnfr.201200522

[4] Jia Z , Sun Y , Yang G , et al. New insights into the PPAR gamma agonists for the treatment of diabetic nephropathy. PPAR Res. 2014;2014:818530. 24624137 10.1155/2014/818530 PMC3927865

[5] Chigurupati S , Dhanaraj SA , Balakumar P . A step ahead of PPARgamma full agonists to PPARgamma partial agonists: therapeutic perspectives in the management of diabetic insulin resistance. Eur J Pharmacol. 2015;755:50‐57. 25748601 10.1016/j.ejphar.2015.02.043

[6] Vallée A , Lecarpentier Y , Guillevin R , Vallee JN . Demyelination in multiple sclerosis: reprogramming energy metabolism and potential PPARgamma agonist treatment approaches. Int J Mol Sci. 2018;19:1212. 10.3390/ijms19041212 PMC5979570 29659554

[7] Zhao L , Yagiz Y , Xu C , Lu J , Chung S , Marshall MR . Muscadine grape seed oil as a novel source of tocotrienols to reduce adipogenesis and adipocyte inflammation. Food Funct. 2015;6:2293‐2302. 26073057 10.1039/c5fo00261c

[8] Nobs SP , Kopf M . PPAR‐gamma in innate and adaptive lung immunity. J Leukoc Biol. 2018;104:737‐741. 29768688 10.1002/JLB.3MR0118-034R

[9] Chung JH , Seo AY , Chung SW , et al. Molecular mechanism of PPAR in the regulation of age‐related inflammation. Ageing Res Rev. 2008;7:126‐136. 18313368 10.1016/j.arr.2008.01.001

[10] Abdelrahman M , Sivarajah A , Thiemermann C . Beneficial effects of PPAR‐gamma ligands in ischemia‐reperfusion injury, inflammation and shock. Cardiovasc Res. 2005;65:772‐781. 15721857 10.1016/j.cardiores.2004.12.008

[11] Giaginis C , Giagini A , Theocharis S . Peroxisome proliferator‐activated receptor‐gamma (PPAR‐gamma) ligands as potential therapeutic agents to treat arthritis. Pharmacol Res. 2009;60:160‐169. 19646655 10.1016/j.phrs.2009.02.005

[12] Staels B . PPARgamma and atherosclerosis. Curr Med Res Opin. 2005;21(suppl 1):S13‐S20. 10.1185/030079905X36440 15811195

[13] Reka AK , Goswami MT , Krishnapuram R , Standiford TJ , Keshamouni VG . Molecular cross‐regulation between PPAR‐gamma and other signaling pathways: implications for lung cancer therapy. Lung Cancer. 2011;72:154‐159. 21354647 10.1016/j.lungcan.2011.01.019 PMC3075310

[14] Lecarpentier Y , Claes V , Vallee A , Hebert JL . Interactions between PPAR gamma and the canonical Wnt/beta‐catenin pathway in type 2 diabetes and colon cancer. PPAR Res. 2017;2017:5879090. 28298922 10.1155/2017/5879090 PMC5337359

[15] Heudobler D , Rechenmacher M , Luke F , et al. Peroxisome proliferator‐activated receptors (PPAR)gamma agonists as master modulators of tumor tissue. Int J Mol Sci. 2018;19:3540. 10.3390/ijms19113540 PMC6274845 30424016

[16] Li H , Wang Q , Dong L , et al. Morusin suppresses breast cancer cell growth in vitro and in vivo through C/EBPbeta and PPARgamma mediated lipoapoptosis. J Exp Clin Cancer Res. 2015;34:137. 26538209 10.1186/s13046-015-0252-4 PMC4634597

[17] Zheng Y , Dai Y , Liu W , et al. Astragaloside IV enhances taxol chemosensitivity of breast cancer via caveolin‐1‐targeting oxidant damage. J Cell Physiol. 2019;234:4277‐4290. 30146689 10.1002/jcp.27196

[18] Vitale G , Zappavigna S , Marra M , et al. The PPAR‐gamma agonist troglitazone antagonizes survival pathways induced by STAT‐3 in recombinant interferon‐beta treated pancreatic cancer cells. Biotechnol Adv. 2012;30:169‐184. 21871555 10.1016/j.biotechadv.2011.08.001

[19] Tan BS , Kang O , Mai CW , et al. 6‐Shogaol inhibits breast and colon cancer cell proliferation through activation of peroxisomal proliferator activated receptor gamma (PPARgamma). Cancer Lett. 2013;336:127‐139. 23612072 10.1016/j.canlet.2013.04.014

[20] Yasui Y , Hosokawa M , Sahara T , et al. Bitter gourd seed fatty acid rich in 9c,11t,13t‐conjugated linolenic acid induces apoptosis and up‐regulates the GADD45, p53 and PPARgamma in human colon cancer Caco‐2 cells. Prostaglandins Leukot Essent Fatty Acids. 2005;73:113‐119. 15961301 10.1016/j.plefa.2005.04.013

[21] Sato H , Ishihara S , Kawashima K , et al. Expression of peroxisome proliferator‐activated receptor (PPAR)gamma in gastric cancer and inhibitory effects of PPARgamma agonists. Br J Cancer. 2000;83:1394‐1400. 11044367 10.1054/bjoc.2000.1457 PMC2408786

[22] Kawai T , Masaki T , Doi S , et al. PPAR‐gamma agonist attenuates renal interstitial fibrosis and inflammation through reduction of TGF‐beta. Lab Invest. 2009;89:47‐58. 19002105 10.1038/labinvest.2008.104

[23] Wei J , Zhu H , Komura K , et al. A synthetic PPAR‐gamma agonist triterpenoid ameliorates experimental fibrosis: PPAR‐gamma‐independent suppression of fibrotic responses. Ann Rheum Dis. 2014;73:446‐454. 23515440 10.1136/annrheumdis-2012-202716 PMC4028127

[24] Kumar V , Mundra V , Mahato RI . Nanomedicines of Hedgehog inhibitor and PPAR‐gamma agonist for treating liver fibrosis. Pharm Res. 2014;31:1158‐1169. 24249038 10.1007/s11095-013-1239-5

[25] Dantas AT , Pereira MC , de Melo Rego MJB , et al. The role of PPAR gamma in systemic sclerosis. PPAR Res. 2015;2015:124624. 26064084 10.1155/2015/124624 PMC4438188

[26] Ichiro Takada MM . PPARg ligands and their therapeutic applications: a patent review (2008–2014). Expert Opin Ther Pat. 2015;25:175‐191. 25416646 10.1517/13543776.2014.985206

[27] Matsuda H , Nakamura S , Yoshikawa M . Search for new type of PPARγ agonist‐like anti‐diabetic compounds from medicinal plants. Biol Pharm Bull. 2014;37:884‐891. 24882400 10.1248/bpb.b14-00037

[28] Wang L , Waltenberger B , Pferschy‐Wenzig EM , et al. Natural product agonists of peroxisome proliferator‐activated receptor gamma (PPARgamma): a review. Biochem Pharmacol. 2014;92:73‐89. 25083916 10.1016/j.bcp.2014.07.018 PMC4212005

[29] del Bas JM , Laos S , Caimari A , Crescenti A , Arola L . Detection of bioavailable peroxisome proliferator‐activated receptor gamma modulators by a cell‐based luciferase reporter system. Anal Biochem. 2012;427:187‐189. 22613053 10.1016/j.ab.2012.05.005

[30] Petersen RK , Christensen KB , Assimopoulou AN , et al. Pharmacophore‐driven identification of PPARgamma agonists from natural sources. J Comput Aided Mol Des. 2011;25:107‐116. 21069556 10.1007/s10822-010-9398-5

[31] Peng YH , Coumar MS , Leou JS , et al. Structural basis for the improved potency of peroxisome proliferator‐activated receptor (PPAR) agonists. ChemMedChem. 2010;5:1707‐1716. 20734309 10.1002/cmdc.201000194

[32] Jang JY , Koh M , Bae H , et al. Structural basis for differential activities of enantiomeric PPARgamma agonists: binding of S35 to the alternate site. Biochim Biophys Acta. 2017;1865:674‐681. 10.1016/j.bbapap.2017.03.008 28342850

[33] Salam NK , Huang TH , Kota BP , Kim MS , Li Y , Hibbs DE . Novel PPAR‐gamma agonists identified from a natural product library: a virtual screening, induced‐fit docking and biological assay study. Chem Biol Drug Des. 2008;71:57‐70. 18086153 10.1111/j.1747-0285.2007.00606.x

[34] Al Sharif M , Alov P , Diukendjieva A , et al. Molecular determinants of PPARgamma partial agonism and related in silico/in vivo studies of natural saponins as potential type 2 diabetes modulators. Food Chem Toxicol. 2018;112:47‐59. 29247773 10.1016/j.fct.2017.12.009

[35] Porskjaer Christensen L , Bahij E‐H . Development of an in vitro screening platform for the identification of partial PPARgamma agonists as a source for antidiabetic lead compounds. Molecules. 2018;23:2431. 10.3390/molecules23102431 PMC6222920 30248999

[36] Maltarollo VG , Togashi M , Nascimento AS , Honorio KM . Structure‐based virtual screening and discovery of New PPARdelta/gamma dual agonist and PPARdelta and gamma agonists. PLoS ONE. 2015;10:e0118790. 25767888 10.1371/journal.pone.0118790 PMC4358979

[37] Vidovic D , Busby SA , Griffin PR , Schurer SC . A combined ligand‐ and structure‐based virtual screening protocol identifies submicromolar PPARgamma partial agonists. ChemMedChem. 2011;6:94‐103. 21162086 10.1002/cmdc.201000428 PMC3517154

[38] El‐Houri RB , Mortier J , Murgueitio MS , Wolber G , Christensen LP . Identification of PPARgamma agonists from natural sources using different in silico approaches. Planta Med. 2015;81:488‐494. 25251562 10.1055/s-0034-1383119

[39] Balakumar P , Rose M , Ganti SS , Krishan P , Singh M . PPAR dual agonists: are they opening Pandora's Box? Pharmacol Res. 2007;56:91‐98. 17428674 10.1016/j.phrs.2007.03.002

[40] Duran‐Sandoval D , Thomas AC , Bailleul B , Fruchart JC , Staels B . Pharmacology of PPARalpha, PPARgamma and dual PPARalpha/gamma agonists in clinical development. Med Sci (Paris). 2003;19:819‐825. 14593612 10.1051/medsci/20031989819

[41] Rubenstrunk A , Hanf R , Hum DW , Fruchart JC , Staels B . Safety issues and prospects for future generations of PPAR modulators. Biochim Biophys Acta. 2007;1771:1065‐1081. 17428730 10.1016/j.bbalip.2007.02.003

[42] Ye G , Chen G , Gao H , et al. Resveratrol inhibits lipid accumulation in the intestine of atherosclerotic mice and macrophages. J Cell Mol Med. 2019;23:4313‐4325. 30957417 10.1111/jcmm.14323 PMC6533483

[43] Hotta M , Nakata R , Katsukawa M , Hori K , Takahashi S , Inoue H . Carvacrol, a component of thyme oil, activates PPARα and γ and suppress COX‐2 expression. J Lipid Res. 2001;51:132‐139. 10.1194/jlr.M900255-JLR200 PMC2789773 19578162

[44] Zhang Y , Xie M , Xue J , Gu Z . Osthole improves fat milk‐induced fatty liver in rats: modulation of hepatic PPAR‐alpha/gamma‐mediated lipogenic gene expression. Planta Med. 2007;73:718‐724. 17611927 10.1055/s-2007-981552

[45] Song LB , Huang JA , Liu ZH , Huang H , Wang KB . Study on activity of dark tea extracts on PPARs model. J Tea Sci. 2008;28:319‐325.

[46] Nobuyuki Takahashi TK , Goto T , Yamamoto T , et al. Dual action of isoprenols from herbal medicines on both PPARγ and PPARu in 3T3‐L1 adipocytes and HepG2 hepatocytes. FEBS Lett. 2002;514:315‐322. 11943173 10.1016/s0014-5793(02)02390-6

[47] Maisa S , Jardat DJN , Baogen WU , Avery MA , Feller DR . Pseudolaric acid analogs as a new class of peroxisome proliferator‐activated receptor agonists. Planta Med. 2002;68:666‐671. 10.1055/s-2002-33785 12221584

[48] Park MY , Lee KS , Sung MK . Effects of dietary mulberry, Korean red ginseng, and banaba on glucose homeostasis in relation to PPAR‐alpha, PPAR‐gamma, and LPL mRNA expressions. Life Sci. 2005;77:3344‐3354. 15979095 10.1016/j.lfs.2005.05.043

[49] O'Sullivan SE . An update on PPAR activation by cannabinoids. Br J Pharmacol. 2016;173:1899‐1910. 27077495 10.1111/bph.13497 PMC4882496

[50] Rogue A , Spire C , Brun M , Claude N , Guillouzo A . Gene expression changes induced by PPAR gamma agonists in animal and human liver. PPAR Res. 2010;2010:325183. 20981297 10.1155/2010/325183 PMC2963138

[51] Wu L , Li J , Liu T , et al. Quercetin shows anti‐tumor effect in hepatocellular carcinoma LM3 cells by abrogating JAK2/STAT3 signaling pathway. Cancer Med. 2019;8:4806‐4820. 31273958 10.1002/cam4.2388 PMC6712453

[52] Wu L , Wang C , Li J , et al. Hepatoprotective effect of quercetin via TRAF6/JNK pathway in acute hepatitis. Biomed Pharmacother. 2017;96:1137‐1146. 29174851 10.1016/j.biopha.2017.11.109

[53] Wu L , Zhang Q , Dai W , et al. Quercetin pretreatment attenuates hepatic ischemia reperfusion‐induced apoptosis and autophagy by inhibiting ERK/NF‐kappaB pathway. Gastroenterol Res Pract. 2017;2017:9724217. 29123547 10.1155/2017/9724217 PMC5662816

[54] Wang W , Wu L , Li J , et al. Alleviation of hepatic ischemia reperfusion injury by oleanolic acid pretreating via reducing HMGB1 release and inhibiting apoptosis and autophagy. Mediators Inflamm. 2019;2019:3240713. 31316298 10.1155/2019/3240713 PMC6604292

[55] Feng J , Wang C , Liu T , et al. Procyanidin B2 inhibits the activation of hepatic stellate cells and angiogenesis via the Hedgehog pathway during liver fibrosis. J Cell Mol Med. 2019;23(9):6479‐6493. 31328391 10.1111/jcmm.14543 PMC6714206

[56] Li S , Wu L , Feng J , et al. In vitro and in vivo study of epigallocatechin‐3‐gallate‐induced apoptosis in aerobic glycolytic hepatocellular carcinoma cells involving inhibition of phosphofructokinase activity. Sci Rep. 2016;6:28479. 27349173 10.1038/srep28479 PMC4923908

[57] Lu X , Liu T , Chen K , et al. Isorhamnetin: a hepatoprotective flavonoid inhibits apoptosis and autophagy via P38/PPAR‐alpha pathway in mice. Biomed Pharmacother. 2018;103:800‐811. 29684859 10.1016/j.biopha.2018.04.016

[58] Li S , Li J , Dai W , et al. Genistein suppresses aerobic glycolysis and induces hepatocellular carcinoma cell death. Br J Cancer. 2017;117:1518‐1528. 28926527 10.1038/bjc.2017.323 PMC5680469

[59] Szeles L , Torocsik D , Nagy L . PPARgamma in immunity and inflammation: cell types and diseases. Biochem Biophys Acta. 2007;1771:1014‐1030. 17418635 10.1016/j.bbalip.2007.02.005

[60] El‐Sheikh AA , Rifaai RA . Peroxisome proliferator activator receptor (PPAR)‐ gamma ligand, but not PPAR‐ alpha, ameliorates cyclophosphamide‐induced oxidative stress and inflammation in rat liver. PPAR Res. 2014;2014:626319. 24803924 10.1155/2014/626319 PMC3996363

[61] Murphy GJ , Holder JC PPAR‐gamma agonists: therapeutic role in diabetes, inflammation and cancer. Trends Pharmacol Sci. 2000;21:469‐474. 11121836 10.1016/s0165-6147(00)01559-5

[62] Croasdell A , Duffney PF , Kim N , Lacy SH , Sime PJ , Phipps RP . PPARgamma and the innate immune system mediate the resolution of inflammation. PPAR Res. 2015;2015:549691. 26713087 10.1155/2015/549691 PMC4680113

[63] Chen K , Li J , Wang J , et al. 15‐Deoxy‐ gamma 12,14‐prostaglandin J2 reduces liver impairment in a model of ConA‐induced acute hepatic inflammation by activation of PPAR gamma and reduction in NF‐ kappa B activity. PPAR Res. 2014;2014:215631. 25120564 10.1155/2014/215631 PMC4121249

[64] Wang F , Liu JC , Zhou RJ , et al. Apigenin protects against alcohol‐induced liver injury in mice by regulating hepatic CYP2E1‐mediated oxidative stress and PPARalpha‐mediated lipogenic gene expression. Chem Biol Interact. 2017;275:171‐177. 28803762 10.1016/j.cbi.2017.08.006

[65] Feng X , Weng D , Zhou F , et al. Activation of PPARgamma by a natural flavonoid modulator, apigenin ameliorates obesity‐related inflammation via regulation of macrophage polarization. EBioMedicine. 2016;9:61‐76. 27374313 10.1016/j.ebiom.2016.06.017 PMC4972579

[66] Mahmoud AM , Mohammed HM , Khadrawy SM , Galaly SR . Hesperidin protects against chemically induced hepatocarcinogenesis via modulation of Nrf2/ARE/HO‐1, PPARgamma and TGF‐beta1/Smad3 signaling, and amelioration of oxidative stress and inflammation. Chem Biol Interact. 2017;277:146‐158. 28935427 10.1016/j.cbi.2017.09.015

[67] Chen X , Ding HW , Li HD , et al. Hesperetin derivative‐14 alleviates inflammation by activating PPAR‐gamma in mice with CCl4‐induced acute liver injury and LPS‐treated RAW264.7 cells. Toxicol Lett. 2017;274:51‐63. 28428136 10.1016/j.toxlet.2017.04.008

[68] El‐Naggar ME , Al‐Joufi F , Anwar M , Attia MF , El‐Bana MA . Curcumin‐loaded PLA‐PEG copolymer nanoparticles for treatment of liver inflammation in streptozotocin‐induced diabetic rats. Colloids Surf B Biointerfaces. 2019;177:389‐398. 30785036 10.1016/j.colsurfb.2019.02.024

[69] Liu S , Su M , Song SJ , Hong J , Chung HY , Jung JH . An anti‐inflammatory PPAR‐gamma agonist from the jellyfish‐derived fungus *Penicillium chrysogenum* J08NF‐4. J Nat Prod. 2018;81:356‐363. 29389121 10.1021/acs.jnatprod.7b00846

[70] Zhang F , Kong D , Lu Y , Zheng S . Peroxisome proliferator‐activated receptor‐gamma as a therapeutic target for hepatic fibrosis: from bench to bedside. Cell Mol Life Sci. 2013;70:259‐276. 22699820 10.1007/s00018-012-1046-x PMC11113701

[71] Zheng S , Chen A . Curcumin suppresses the expression of extracellular matrix genes in activated hepatic stellate cells by inhibiting gene expression of connective tissue growth factor. Am J Physiol Gastrointest Liver Physiol. 2006;290:G883‐G893. 16306131 10.1152/ajpgi.00450.2005

[72] Zheng S , Chen A . Disruption of transforming growth factor‐beta signaling by curcumin induces gene expression of peroxisome proliferator‐activated receptor in rat hepatic stellate cells. Am J Physiol Gastrointest Liver Physiol. 2006;292:G113‐G123. 16959952 10.1152/ajpgi.00200.2006

[73] Guo C , Xu L , He Q , Liang T , Duan X , Li R . Anti‐fibrotic effects of puerarin on CCl4‐induced hepatic fibrosis in rats possibly through the regulation of PPAR‐gamma expression and inhibition of PI3K/Akt pathway. Food Chem Toxicol. 2013;56:436‐442. 23500774 10.1016/j.fct.2013.02.051

[74] Hsu WH , Lee BH , Hsu YW , Pan TM . Peroxisome proliferator‐activated receptor‐gamma activators monascin and rosiglitazone attenuate carboxymethyllysine‐induced fibrosis in hepatic stellate cells through regulating the oxidative stress pathway but independent of the receptor for advanced glycation end products signaling. J Agric Food Chem. 2013;61:6873‐6879. 23796251 10.1021/jf402082g

[75] Choi JH , Jin SW , Choi CY , et al. Capsaicin inhibits dimethylnitrosamine‐induced hepatic fibrosis by inhibiting the TGF‐beta1/Smad pathway via peroxisome proliferator‐activated receptor gamma activation. J Agric Food Chem. 2017;65:317‐326. 27991776 10.1021/acs.jafc.6b04805

[76] Hongwen Ma HWS , Kolattukudy PE . Estrogen‐induced production of a peroxisome proliferator‐activated receptor (PPAR) ligand in a PPAR‐expressing tissue. J Biol Chem. 1998;273:30131‐30138. 9804768 10.1074/jbc.273.46.30131

[77] Borbath I , Horsmans Y . The role of PPARgamma in hepatocellular carcinoma. PPAR Res. 2008;2008:209520. 18509497 10.1155/2008/209520 PMC2396389

[78] Veliceasa D , Schulze‐Hoepfner FT , Volpert OV . PPARgamma and agonists against cancer: rational design of complementation treatments. PPAR Res. 2008;2008:945275. 19043603 10.1155/2008/945275 PMC2586323

[79] Koga H , Sakisaka S , Harada M , et al. Involvement of p21(WAF1/Cip1), p27(Kip1), and p18(INK4c) in troglitazone‐induced cell‐cycle arrest in human hepatoma cell lines. Hepatology. 2001;33:1087‐1097. 11343236 10.1053/jhep.2001.24024

[80] Katherine L , Schaefer KW , Takahashi H , et al. Peroxisome proliferator‐activated receptor; inhibition prevents adhesion to the extracellular matrix and induces anoikis in hepatocellular carcinoma cells. Cancer Res. 2005;65:2251‐2259. 15781638 10.1158/0008-5472.CAN-04-3037

[81] Lin YM , Velmurugan BK , Yeh YL , et al. Activation of estrogen receptors with E2 downregulates peroxisome proliferator‐activated receptor gamma in hepatocellular carcinoma. Oncol Rep. 2013;30:3027‐3031. 24126791 10.3892/or.2013.2793

[82] Yu J , Qiao L , Zimmermann L , et al. Troglitazone inhibits tumor growth in hepatocellular carcinoma in vitro and in vivo. Hepatology. 2006;43:134‐143. 16374840 10.1002/hep.20994

[83] Ming‐Yi Li HD , Zhao J‐M , Dai D , Xiao‐Yu T . Peroxisome proliferator‐activated receptor gamma ligands inhibit cell growth and induce apoptosis in human liver cancer BEL‐7402 cells. World J Gastroenterol. 2003;9:1683‐1688. 12918101 10.3748/wjg.v9.i8.1683 PMC4611524

[84] Toyoda M , Takagi H , Horiguchi N , et al. A ligand for peroxisome proliferator activated receptor gamma inhibits cell growth and induces apoptosis in human liver cancer cells. Gut. 2002;50:563‐567. 11889080 10.1136/gut.50.4.563 PMC1773180

[85] Rumi MA , Sato H , Ishihara S , et al. Peroxisome proliferator‐activated receptor gamma ligand‐induced growth inhibition of human hepatocellular carcinoma. Br J Cancer. 2001;84:1640‐1647. 11401318 10.1054/bjoc.2001.1821 PMC2363681

[86] Koga H , Harada M , Ohtsubo M , et al. Troglitazone induces p27Kip1‐associated cell‐cycle arrest through down‐regulating Skp2 in human hepatoma cells. Hepatology. 2003;37:1086‐1096. 12717389 10.1053/jhep.2003.50186

[87] Zhou YM , Wen YH , Kang XY , Qian HH , Yang JM , Yin ZF . Troglitazone, a peroxisome proliferator‐activated receptor gamma ligand, induces growth inhibition and apoptosis of HepG2 human liver cancer cells. World J Gastroenterol. 2008;14:2168‐2173. 18407589 10.3748/wjg.14.2168 PMC2703840

[88] Wang Z , Li F , Quan Y , Shen J . Avicularin ameliorates human hepatocellular carcinoma via the regulation of NFkappaB/COX2/PPARgamma activities. Mol Med Rep. 2019;19:5417‐5423. 31059053 10.3892/mmr.2019.10198 PMC6522888

[89] Han M , Gao H , Ju P , et al. Hispidulin inhibits hepatocellular carcinoma growth and metastasis through AMPK and ERK signaling mediated activation of PPARgamma. Biomed Pharmacother. 2018;103:272‐283. 29656183 10.1016/j.biopha.2018.04.014

[90] Huang C , Wei Y‐X , Shen M‐C , Tu Y‐H , Wang C‐C , Huang H‐C . Chrysin, abundant in morinda citrifolia fruit water–EtOAc extracts, combined with apigenin synergistically induced apoptosis and inhibited migration in human breast and liver cancer cells. J Agric Food Chem. 2016;64:4235‐4245. 27137679 10.1021/acs.jafc.6b00766

[91] Vara D , Morell C , Rodriguez‐Henche N , Diaz‐Laviada I . Involvement of PPARgamma in the antitumoral action of cannabinoids on hepatocellular carcinoma. Cell Death Dis. 2013;4:e618. 23640460 10.1038/cddis.2013.141 PMC3674350

[92] Zhang X , Zhao WE , Hu L , Zhao L , Huang J . Carotenoids inhibit proliferation and regulate expression of peroxisome proliferators‐activated receptor gamma (PPARgamma) in K562 cancer cells. Arch Biochem Biophys. 2011;512:96‐106. 21620794 10.1016/j.abb.2011.05.004

[93] Zurlo D , Assante G , Moricca S , Colantuoni V , Lupo A , Cladosporol A . a new peroxisome proliferator‐activated receptor gamma (PPARgamma) ligand, inhibits colorectal cancer cells proliferation through beta‐catenin/TCF pathway inactivation. Biochem Biophys Acta. 2014;1840:2361‐2372. 24735796 10.1016/j.bbagen.2014.04.007

[94] Kiskova T , Kassayova M . Resveratrol action on lipid metabolism in cancer. Int J Mol Sci. 2019;20(11):2704. 10.3390/ijms20112704 PMC6601040 31159437

[95] Ables GP . Update on ppargamma and nonalcoholic fatty liver disease. PPAR Res. 2012;2012:912351. 22966224 10.1155/2012/912351 PMC3431124

[96] Skat‐Rordam J , Hojland Ipsen D , Lykkesfeldt J , Tveden‐Nyborg P . A role of peroxisome proliferator‐activated receptor gamma in non‐alcoholic fatty liver disease. Basic Clin Pharmacol Toxicol. 2019;124:528‐537. 30561132 10.1111/bcpt.13190 PMC6850367

[97] Chinetti G , Fruchart J‐C , Staels B . Peroxisome proliferator‐activated receptors (PPARs): Nuclear receptors at the crossroads between lipid metabolism and inflammation. Inflamm Res. 2000;49:497‐505. 11089900 10.1007/s000110050622

[98] Nan YM , Fu N , Wu WJ , et al. Rosiglitazone prevents nutritional fibrosis and steatohepatitis in mice. Scand J Gastroenterol. 2009;44:358‐365. 18991162 10.1080/00365520802530861

[99] Camirand A , Marie V , Rabelo R , Silva JE . Thiazolidinediones stimulate uncoupling protein‐2 expression in cell lines representing white and brown adipose tissues and skeletal muscle. Endocrinology. 1998;139:428‐431. 9421444 10.1210/endo.139.1.5808

[100] Wu Z , Xie Y , Morrison RF , Bucher NL , Farmer SR . PPARγ induces the insulin‐dependent glucose transporter GLUT4 in the absence of C/EBPα during the conversion of 3T3 fibroblasts into adipocytes. J Clin Invest. 1998;101:22‐23. 9421462 10.1172/JCI1244 PMC508536

[101] Malloy VL , Krajcik RA , Bailey SJ , Hristopoulos G , Plummer JD , Orentreich N . Methionine restriction decreases visceral fat mass and preserves insulin action in aging male Fischer 344 rats independent of energy restriction. Aging Cell. 2006;5:305‐314. 16800846 10.1111/j.1474-9726.2006.00220.x

[102] Sun X , Tang Y , Tan X , et al. Activation of peroxisome proliferator‐activated receptor‐gamma by rosiglitazone improves lipid homeostasis at the adipose tissue‐liver axis in ethanol‐fed mice. Am J Physiol Gastrointest Liver Physiol. 2012;302:G548‐G557. 22173916 10.1152/ajpgi.00342.2011 PMC3311430

[103] Vidal‐Puig A , Jimenez‐Linan M , Lowell BB , et al. Regulation of PPAR gamma gene expression by nutrition and obesity in rodents. J Clin Invest. 1996;97:2553‐2561. 8647948 10.1172/JCI118703 PMC507341

[104] Moran‐Salvador E , Lopez‐Parra M , Garcia‐Alonso V , et al. Role for PPARgamma in obesity‐induced hepatic steatosis as determined by hepatocyte‐ and macrophage‐specific conditional knockouts. FASEB J. 2011;25:2538‐2550. 21507897 10.1096/fj.10-173716

[105] Uno K , Katagiri H , Yamada T , et al. Neuronal pathway from the liver modulates energy expenditure and systemic insulin sensitivity. Science. 2006;312:1656‐1659. 16778057 10.1126/science.1126010

[106] Gavrilova O , Haluzik M , Matsusue K , et al. Liver peroxisome proliferator‐activated receptor gamma contributes to hepatic steatosis, triglyceride clearance, and regulation of body fat mass. J Biol Chem. 2003;278:34268‐34276. 12805374 10.1074/jbc.M300043200

[107] Orsolya Mezei WJB , Steger RW , Peluso MR , Winters TA , Shay N . Soy isoflavones exert antidiabetic and hypolipidemic effects through the PPAR pathways in obese Zucker rats and murine RAW 264.7 cells. J Nutr. 2002;133:1238‐1243. 10.1093/jn/133.5.1238 12730403

[108] Andrade JM , Paraiso AF , de Oliveira MV , et al. Resveratrol attenuates hepatic steatosis in high‐fat fed mice by decreasing lipogenesis and inflammation. Nutrition. 2014;30:915‐919. 24985011 10.1016/j.nut.2013.11.016

[109] Dominguez‐Avila JA , Gonzalez‐Aguilar GA , Alvarez‐Parrilla E , de la Rosa LA . Modulation of expression and activity in response to polyphenolic compounds in high fat diets. Int J Mol Sci. 2016;17:1002. 10.3390/ijms17071002 PMC4964378 27367676

[110] Feng L , Luo H , Xu Z , et al. Bavachinin, as a novel natural pan‐PPAR agonist, exhibits unique synergistic effects with synthetic PPAR‐gamma and PPAR‐alpha agonists on carbohydrate and lipid metabolism in db/db and diet‐induced obese mice. Diabetologia. 2016;59:1276‐1286. 26983922 10.1007/s00125-016-3912-9

[111] Barone R , Rizzo R , Tabbi G , Malaguarnera M , Frye RE , Bastin J . Nuclear peroxisome proliferator‐activated receptors (PPARs) as therapeutic targets of resveratrol for autism spectrum disorder. Int J Mol Sci. 2019;20:1878. 10.3390/ijms20081878 PMC6515064 30995737

[112] Serra D , Almeida LM , Dinis TC . Anti‐inflammatory protection afforded by cyanidin‐3‐glucoside and resveratrol in human intestinal cells via Nrf2 and PPAR‐gamma: Comparison with 5‐aminosalicylic acid. Chem Biol Interact. 2016;260:102‐109. 27818126 10.1016/j.cbi.2016.11.003

[113] Regnault TR , Zhao L , Chiu JS , Gottheil SK , Foran A , Yee SP . Peroxisome proliferator‐activated receptor ‐beta/delta, ‐gamma agonists and resveratrol modulate hypoxia induced changes in nuclear receptor activators of muscle oxidative metabolism. PPAR Res. 2010;2010:129173. 21113404 10.1155/2010/129173 PMC2991640

[114] Said RS , El‐Demerdash E , Nada AS , Kamal MM . Resveratrol inhibits inflammatory signaling implicated in ionizing radiation‐induced premature ovarian failure through antagonistic crosstalk between silencing information regulator 1 (SIRT1) and poly(ADP‐ribose) polymerase 1 (PARP‐1). Biochem Pharmacol. 2016;103:140‐150. 26827941 10.1016/j.bcp.2016.01.019

[115] Jagtap S , Meganathan K , Wagh V , Winkler J , Hescheler J , Sachinidis A . Chemoprotective mechanism of the natural compounds, epigallocatechin‐3‐O‐gallate, quercetin and curcumin against cancer and cardiovascular diseases. Curr Med Chem. 2009;16:1451‐1462. 19355899 10.2174/092986709787909578

[116] Chen A , Zheng S . Curcumin inhibits connective tissue growth factor gene expression in activated hepatic stellate cells in vitro by blocking NF‐kappaB and ERK signalling. Br J Pharmacol. 2008;153:557‐567. 17965732 10.1038/sj.bjp.0707542 PMC2241795

[117] Miao M , Liu J , Wang T , Liang X , Bai M . The effect of different proportions of astragaloside and curcumin on DM model of mice. Saudi Pharm J. 2017;25:477‐481. 28579878 10.1016/j.jsps.2017.04.009 PMC5447424

[118] Arzuman L , Beale P , Chan C , Yu JQ , Huq F . Synergism from combinations of tris(benzimidazole) monochloroplatinum(II) chloride with capsaicin, quercetin, curcumin and cisplatin in human ovarian cancer cell lines. Anticancer Res. 2014;34:5453‐5464. 25275041

[119] Nishiyama TMT , Kishida H , Tsukagawa M , et al. Curcuminoids and sesquiterpenoids in turmeric (*Curcuma longa* L.) suppress an increase in blood glucose level in type 2 diabetic KK‐Ay mice. J Agric Food Chem. 2005;53:959‐963. 15713005 10.1021/jf0483873

[120] Sakamoto Y , Kanatsu J , Toh M , Naka A , Kondo K , Iida K . The dietary isoflavone daidzein reduces expression of pro‐inflammatory genes through PPARalpha/gamma and JNK pathways in adipocyte and macrophage co‐cultures. PLoS ONE. 2016;11:e0149676. 26901838 10.1371/journal.pone.0149676 PMC4763373

[121] Anandharajan R , Pathmanathan K , Shankernarayanan NP , Vishwakarma RA , Balakrishnan A . Upregulation of Glut‐4 and PPAR gamma by an isoflavone from *Pterocarpus marsupium* on L6 myotubes: a possible mechanism of action. J Ethnopharmacol. 2005;97:253‐260. 15707762 10.1016/j.jep.2004.11.023

[122] Sun Y , Bennett A . Cannabinoids: a new group of agonists of PPARs. PPAR Res. 2007;2007:23513. 18288264 10.1155/2007/23513 PMC2220031

[123] Campos AC , Fogaca MV , Sonego AB , Guimaraes FS . Cannabidiol, neuroprotection and neuropsychiatric disorders. Pharmacol Res. 2016;112:119‐127. 26845349 10.1016/j.phrs.2016.01.033

[124] dos‐Santos‐Pereira M , da‐Silva CA , Guimarães FS , Del‐Bel E . Co‐administration of cannabidiol and capsazepine reduces L‐DOPA‐induced dyskinesia in mice: possible mechanism of action. Neurobiol Dis. 2016;94:179‐195. 27373843 10.1016/j.nbd.2016.06.013

[125] Hegde VL , Singh UP , Nagarkatti PS , Nagarkatti M . Critical role of mast cells and peroxisome proliferator‐activated receptor gamma in the induction of myeloid‐derived suppressor cells by marijuana cannabidiol in vivo. J Immunol. 2015;194:5211‐5222. 25917103 10.4049/jimmunol.1401844 PMC4433789

[126] Moldzio R , Pacher T , Krewenka C , et al. Effects of cannabinoids Delta(9)‐tetrahydrocannabinol, Delta(9)‐tetrahydrocannabinolic acid and cannabidiol in MPP+ affected murine mesencephalic cultures. Phytomedicine. 2012;19:819‐824. 22571976 10.1016/j.phymed.2012.04.002

[127] Ibeas Bih C , Chen T , Nunn AV , Bazelot M , Dallas M , Whalley BJ . Molecular targets of cannabidiol in neurological disorders. Neurotherapeutics. 2015;12:699‐730. 26264914 10.1007/s13311-015-0377-3 PMC4604182

[128] Perez M , Cartarozzi LP , Chiarotto GB , Oliveira SA , Guimaraes FS , Oliveira ALR . Neuronal preservation and reactive gliosis attenuation following neonatal sciatic nerve axotomy by a fluorinated cannabidiol derivative. Neuropharmacology. 2018;140:201‐208. 30096328 10.1016/j.neuropharm.2018.08.009

[129] Onofri C , de Meijer EPM , Mandolino G . Sequence heterogeneity of cannabidiolic‐ and tetrahydrocannabinolic acid‐synthase in *Cannabis sativa* L. and its relationship with chemical phenotype. Phytochemistry. 2015;116:57‐68. 25865737 10.1016/j.phytochem.2015.03.006

[130] Ebbert JO , Scharf EL , Hurt RT . Medical cannabis. Mayo Clin Proc. 2018;93:1842‐1847. 30522595 10.1016/j.mayocp.2018.09.005

[131] Allsop DJ , Lintzeris N , Copeland J , Dunlop A , McGregor IS . Cannabinoid replacement therapy (CRT): nabiximols (Sativex) as a novel treatment for cannabis withdrawal. Clin Pharmacol Ther. 2015;97:571‐574. 25777582 10.1002/cpt.109

[132] Castelli L , Prosperini L , Pozzilli C . Balance worsening associated with nabiximols in multiple sclerosis. Mult Scler. 2019;25:113‐117. 29533137 10.1177/1352458518765649

[133] Riva N , Mora G , Sorarù G , et al. Safety and efficacy of nabiximols on spasticity symptoms in patients with motor neuron disease (CANALS): a multicentre, double‐blind, randomised, placebo‐controlled, phase 2 trial. Lancet Neurol. 2019;18:155‐164. 30554828 10.1016/S1474-4422(18)30406-X

[134] Sorosina M , Clarelli F , Ferre L , et al. Clinical response to Nabiximols correlates with the downregulation of immune pathways in multiple sclerosis. Eur J Neurol. 2018;25:934‐e70. 29528549 10.1111/ene.13623

[135] Kwok H‐H , Guo G‐L , Lau J‐C , et al. Stereoisomers ginsenosides‐20(S)‐Rg(3) and ‐20(R)‐Rg(3) differentially induce angiogenesis through peroxisome proliferator‐activated receptor‐gamma. Biochem Pharmacol. 2012;83:893‐902. 22234331 10.1016/j.bcp.2011.12.039

[136] Puhl AC , Bernardes A , Silveira RL , et al. Mode of peroxisome proliferator‐activated receptor gamma activation by luteolin. Mol Pharmacol. 2012;81:788‐799. 22391103 10.1124/mol.111.076216

[137] El‐Bassossy HM , Abo‐Warda SM , Fahmy A . Chrysin and luteolin alleviate vascular complications associated with insulin resistance mainly through PPAR‐gamma activation. Am J Chin Med. 2014;42:1153‐1167. 25169908 10.1142/S0192415X14500724

[138] Zhang ML , Irwin D , Li XN , Sauriol F , Shi XW . PPARgamma agonist from *Chromolaena odorata* . J Nat Prod. 2012;75:2076‐2081. 23186307 10.1021/np300386d

[139] Dat NT , Lee K , Hong YS , Kim YH , Minh CV , Lee JJ . A peroxisome proliferator‐activated receptor‐gamma agonist and other constituents from *Chromolaena odorata* . Planta Med. 2009;75:803‐807. 19242902 10.1055/s-0029-1185386

[140] Mahmoud AM , Abdella EM , El‐Derby AM , Abdella EM . Protective effects of *Turbinaria ornata* and *Padina pavonica* against azoxymethane‐induced colon carcinogenesis through modulation of PPAR gamma, NF‐kappaB and oxidative stress. Phytother Res. 2015;29:737‐748. 25676613 10.1002/ptr.5310

[141] Jin YG , Yuan Y , Wu QQ , et al. Puerarin protects against cardiac fibrosis associated with the inhibition of TGF‐beta1/Smad2‐mediated endothelial‐to‐mesenchymal transition. PPAR Res. 2017;2017:2647129. 28638404 10.1155/2017/2647129 PMC5468594

[142] Ma ZG , Yuan YP , Zhang X , Xu SC , Wang SS , Tang QZ . Piperine attenuates pathological cardiac fibrosis via PPAR‐gamma/AKT pathways. EBioMedicine. 2017;18:179‐187. 28330809 10.1016/j.ebiom.2017.03.021 PMC5405163

[143] Guan C , Qiao S , Lv Q , et al. Orally administered berberine ameliorates bleomycin‐induced pulmonary fibrosis in mice through promoting activation of PPAR‐gamma and subsequent expression of HGF in colons. Toxicol Appl Pharmacol. 2018;343:1‐15. 29408570 10.1016/j.taap.2018.02.001

[144] Hsu WH , Lee BH , Pan TM . Monascin attenuates oxidative stress‐mediated lung inflammation via peroxisome proliferator‐activated receptor‐gamma (PPAR‐gamma) and nuclear factor‐erythroid 2 related factor 2 (Nrf‐2) modulation. J Agric Food Chem. 2014;62:5337‐5344. 24865672 10.1021/jf501373a

[145] Lee BH , Hsu WH , Liao TH , Pan TM . The Monascus metabolite monascin against TNF‐alpha‐induced insulin resistance via suppressing PPAR‐gamma phosphorylation in C2C12 myotubes. Food Chem Toxicol. 2011;49:2609‐2617. 21777645 10.1016/j.fct.2011.07.005

[146] Hsu W‐H , Pan T‐M . A novel PPARgamma agonist monascin's potential application in diabetes prevention. Food Funct. 2014;5:1334‐1440. 24752777 10.1039/c3fo60575b

[147] Pelham CJ , Ketsawatsomkron P , Groh S , et al. Cullin‐3 regulates vascular smooth muscle function and arterial blood pressure via PPARgamma and RhoA/Rho‐kinase. Cell Metab. 2012;16:462‐472. 23040068 10.1016/j.cmet.2012.08.011 PMC3474846

[148] Lee BH , Hsu WH , Chang YY , Kuo HF , Hsu YW , Pan TM . Ankaflavin: a natural novel PPARgamma agonist upregulates Nrf2 to attenuate methylglyoxal‐induced diabetes in vivo. Free Radic Biol Med. 2012;53:2008‐2016. 23022408 10.1016/j.freeradbiomed.2012.09.025

[149] Inoue M , Tanabe H , Matsumoto A , et al. Astaxanthin functions differently as a selective peroxisome proliferator‐activated receptor gamma modulator in adipocytes and macrophages. Biochem Pharmacol. 2012;84:692‐700. 22732454 10.1016/j.bcp.2012.05.021

[150] Qiu L , Lin B , Lin Z , Lin Y , Lin M , Yang X . Biochanin A ameliorates the cytokine secretion profile of lipopolysaccharide‐stimulated macrophages by a PPARgamma‐dependent pathway. Mol Med Rep. 2012;5:217‐222. 21946955 10.3892/mmr.2011.599

[151] Liu H , Wang S , Sun A , et al. Danhong inhibits oxidized low‐density lipoprotein‐induced immune maturation of dentritic cells via a peroxisome proliferator activated receptor gamma‐mediated pathway. J Pharmacol Sci. 2012;119:1‐9. 22739234 10.1254/jphs.11226fp

[152] Hurtado O , Ballesteros I , Cuartero MI , et al. Daidzein has neuroprotective effects through ligand‐binding‐independent PPARgamma activation. Neurochem Int. 2012;61:119‐127. 22521773 10.1016/j.neuint.2012.04.007

[153] Liu Y , Zhao B , Mao G , et al. Epigallocatechin‐3‐O‐gallate, a green tea polyphenol, induces expression of pim‐1 kinase via PPARgamma in human vascular endothelial cells. Cardiovasc Toxicol. 2013;13:391‐395. 23990052 10.1007/s12012-013-9220-4

[154] Kim SH , Hong JH , Lee YC . Ursolic acid, a potential PPARgamma agonist, suppresses ovalbumin‐induced airway inflammation and Penh by down‐regulating IL‐5, IL‐13, and IL‐17 in a mouse model of allergic asthma. Eur J Pharmacol. 2013;701:131‐143. 23201068 10.1016/j.ejphar.2012.11.033

[155] Lee BH , Hsu WH , Huang T , Chang YY , Hsu YW , Pan TM . Monascin improves diabetes and dyslipidemia by regulating PPARgamma and inhibiting lipogenesis in fructose‐rich diet‐induced C57BL/6 mice. Food Funct. 2013;4:950‐959. 23673903 10.1039/c3fo60062a

[156] Rani N , Bharti S , Bhatia J , et al. Inhibition of TGF‐beta by a novel PPAR‐gamma agonist, chrysin, salvages beta‐receptor stimulated myocardial injury in rats through MAPKs‐dependent mechanism. Nutr Metab (Lond). 2015;12:11. 25774203 10.1186/s12986-015-0004-7 PMC4359541

[157] Jin Y , Liu K , Peng J , et al. Rhizoma dioscoreae nipponicae polysaccharides protect HUVECs from H_2_O_2_‐induced injury by regulating PPARgamma factor and the NADPH oxidase/ROS‐NF‐kappaB signal pathway. Toxicol Lett. 2015;232:149‐158. 25305479 10.1016/j.toxlet.2014.10.006

[158] Kim TK , Park KS . Inhibitory effects of harpagoside on TNF‐alpha‐induced pro‐inflammatory adipokine expression through PPAR‐gamma activation in 3T3‐L1 adipocytes. Cytokine. 2015;76:368‐374. 26049170 10.1016/j.cyto.2015.05.015

[159] Li QY , Chen L , Yan MM , Shi XJ , Zhong MK . Tectorigenin regulates adipogenic differentiation and adipocytokines secretion via PPARgamma and IKK/NF‐kappaB signaling. Pharm Biol. 2015;53:1567‐1575. 25856699 10.3109/13880209.2014.993038

[160] Rani N , Bharti S , Bhatia J , Nag TC , Ray R , Arya DS . Chrysin, a PPAR‐gamma agonist improves myocardial injury in diabetic rats through inhibiting AGE‐RAGE mediated oxidative stress and inflammation. Chem Biol Interact. 2016;250:59‐67. 26972669 10.1016/j.cbi.2016.03.015

[161] Ge J , Miao JJ , Sun XY , Yu JY . Huangkui capsule, an extract from *Abelmoschus manihot* (L.) medic, improves diabetic nephropathy via activating peroxisome proliferator‐activated receptor (PPAR)‐alpha/gamma and attenuating endoplasmic reticulum stress in rats. J Ethnopharmacol. 2016;189:238‐249. 27224243 10.1016/j.jep.2016.05.033

[162] Lin N , Chen LM , Pan XD , et al. Tripchlorolide attenuates beta‐amyloid generation via suppressing PPARgamma‐regulated BACE1 activity in N2a/APP695 cells. Mol Neurobiol. 2016;53:6397‐6406. 26582466 10.1007/s12035-015-9542-2

[163] Jeon H , Kim DH , Nho YH , Park JE , Kim SN , Choi EH . A mixture of extracts of *Kochia scoparia* and *Rosa multiflora* with PPAR alpha/gamma dual agonistic effects prevents photoaging in hairless mice. Int J Mol Sci. 2016;17:1919. 10.3390/ijms17111919 PMC5133916 27854351

[164] Zhang ZX , Li YB , Zhao RP . Epigallocatechin gallate attenuates beta‐amyloid generation and oxidative stress involvement of PPARgamma in N2a/APP695 cells. Neurochem Res. 2017;42:468‐480. 27889855 10.1007/s11064-016-2093-8

[165] Tsujino Y . A new agonist for peroxisome proliferation‐activated receptor gamma (PPARgamma), Fraglide‐1 from Zhenjiang fragrant vinegar: screening and characterization based on cell culture experiments. J Oleo Sci. 2017;66:615‐622. 28515383 10.5650/jos.ess16253

[166] Xu X , Wang Y , Wei Z , et al. Madecassic acid, the contributor to the anti‐colitis effect of madecassoside, enhances the shift of Th17 toward Treg cells via the PPARgamma/AMPK/ACC1 pathway. Cell Death Dis. 2017;8:e2723. 28358365 10.1038/cddis.2017.150 PMC5386545

[167] Ke B , Ke X , Wan X , et al. Astragalus polysaccharides attenuates TNF‐alpha‐induced insulin resistance via suppression of miR‐721 and activation of PPAR‐gamma and PI3K/AKT in 3T3‐L1 adipocytes. Am J Transl Res. 2017;9:2195‐2206. 28559971 PMC5446503

[168] Xu LJ , Yu MH , Huang CY , et al. Isoprenylated flavonoids from *Morus nigra *and their PPAR gamma agonistic activities. Fitoterapia. 2018;127:109‐114. 29427594 10.1016/j.fitote.2018.02.004

[169] Bhat OM , Kumar PU , Rao KR , Ahmad A , Dhawan V . Terminalia arjuna prevents interleukin‐18‐induced atherosclerosis via modulation of NF‐kappaB/PPAR‐gamma‐mediated pathway in Apo E‐/‐ mice. Inflammopharmacology. 2018;26:583‐598. 28547701 10.1007/s10787-017-0357-9

[170] Kong R , Luo H , Wang N , et al. Portulaca extract attenuates development of dextran sulfate sodium induced colitis in mice through activation of PPARgamma. PPAR Res. 2018;2018:6079101. 29483924 10.1155/2018/6079101 PMC5816873

[171] Xu GM , Zan T , Li HY , et al. Betulin inhibits lipopolysaccharide/D‐galactosamine‐induced acute liver injury in mice through activating PPAR‐gamma. Biomed Pharmacother. 2018;106:941‐945. 30119266 10.1016/j.biopha.2018.07.011

[172] Cui J , Wang G , Kandhare AD , Mukherjee‐Kandhare AA , Bodhankar SL . Neuroprotective effect of naringin, a flavone glycoside in quinolinic acid‐induced neurotoxicity: possible role of PPAR‐gamma, Bax/Bcl‐2, and caspase‐3. Food Chem Toxicol. 2018;121:95‐108. 30130594 10.1016/j.fct.2018.08.028

[173] Youssef DA , El‐Fayoumi HM , Mahmoud MF . Beta‐caryophyllene protects against diet‐induced dyslipidemia and vascular inflammation in rats: Involvement of CB2 and PPAR‐gamma receptors. Chem Biol Interact. 2019;297:16‐24. 30343038 10.1016/j.cbi.2018.10.010

[174] Badawy AM , El‐Naga RN , Gad AM , Tadros MG , Fawzy HM . Wogonin pre‐treatment attenuates cisplatin‐induced nephrotoxicity in rats: Impact on PPAR‐gamma, inflammation, apoptosis and Wnt/beta‐catenin pathway. Chem Biol Interact. 2019;308:137‐146. 31103702 10.1016/j.cbi.2019.05.029

[175] Meng HW , You HM , Yang Y , et al. 4‐Methylcoumarin‐[5,6‐g]‐hesperetin attenuates inflammatory responses in alcoholic hepatitis through PPAR‐gamma activation. Toxicology. 2019;421:9‐21. 30951781 10.1016/j.tox.2019.04.004

[176] Wakabayashi K , Hayashi S , Matsui Y , et al. Pharmacology and in vitro profiling of a novel peroxisome proliferator‐activated receptor gamma ligand, Cerco‐A. Biol Pharm Bull. 2011;34:1094‐1104. 21720019 10.1248/bpb.34.1094

[177] Kwon DY , Kim DS , Yang HJ , Park S . The lignan‐rich fractions of Fructus Schisandrae improve insulin sensitivity via the PPAR‐gamma pathways in in vitro and in vivo studies. J Ethnopharmacol. 2011;135:455‐462. 21440615 10.1016/j.jep.2011.03.037

[178] Xu Y , Lai F , Xu Y , et al. Mycophenolic acid induces ATP‐binding cassette transporter A1 (ABCA1) expression through the PPARgamma‐LXRalpha‐ABCA1 pathway. Biochem Biophys Res Commun. 2011;414:779‐782. 22005457 10.1016/j.bbrc.2011.10.002

[179] Weidner C , Wowro SJ , Freiwald A , et al. Amorfrutin B is an efficient natural peroxisome proliferator‐activated receptor gamma (PPARgamma) agonist with potent glucose‐lowering properties. Diabetologia. 2013;56:1802‐1812. 23680913 10.1007/s00125-013-2920-2

[180] Atanasov AG , Wang JN , Gu SP , et al. Honokiol: a non‐adipogenic PPARgamma agonist from nature. Biochem Biophys Acta. 2013;1830:4813‐4819. 23811337 10.1016/j.bbagen.2013.06.021 PMC3790966

[181] Shyni GL , Kavitha S , Indu S , et al. Chebulagic acid from *Terminalia chebula* enhances insulin mediated glucose uptake in 3T3‐L1 adipocytes via PPARgamma signaling pathway. BioFactors. 2014;40:646‐657. 25529897 10.1002/biof.1193

[182] Beekmann K , Rubió L , de Haan LH . The effect of quercetin and kaempferol aglycones and glucuronides on peroxisome proliferator‐activated receptor‐gamma (PPAR‐gamma). Food Funct. 2015;6:1098‐1107. 25765892 10.1039/c5fo00076a

[183] Han JM , Kim MH , Choi YY , Lee H , Hong J , Yang WM . Effects of *Lonicera japonica* Thunb. on type 2 diabetes via PPAR‐gamma activation in rats. Phytotherapy Res. 2015;29:1616‐1621. 10.1002/ptr.5413 26174209

[184] Zhang Y , Gu M , Cai W , et al. Dietary component isorhamnetin is a PPARgamma antagonist and ameliorates metabolic disorders induced by diet or leptin deficiency. Sci Rep. 2016;6:19288. 26775807 10.1038/srep19288 PMC4726074

[185] Montanari R , Capelli D , Tava A , et al. Screening of saponins and sapogenins from Medicago species as potential PPARgamma agonists and X‐ray structure of the complex PPARgamma/caulophyllogenin. Sci Rep. 2016;6:27658. 27283034 10.1038/srep27658 PMC4901321

[186] Kunasegaran T , Mustafa MR , Murugan DD , Achike FI . The bioflavonoid quercetin synergises with PPAR‐gamma agonist pioglitazone in reducing angiotensin‐II contractile effect in fructose‐streptozotocin induced diabetic rats. Biochimie. 2016;125:131‐139. 27012965 10.1016/j.biochi.2016.03.008

[187] Qi ZG , Zhao X , Zhong W , Xie ML . Osthole improves glucose and lipid metabolism via modulation of PPARalpha/gamma‐mediated target gene expression in liver, adipose tissue, and skeletal muscle in fatty liver rats. Pharm Biol. 2016;54:882‐888. 26455539 10.3109/13880209.2015.1089295

[188] Wang X , Wang Y , Hu JP , et al. Astragaloside IV, a natural PPARgamma agonist, reduces abeta production in Alzheimer's disease through inhibition of BACE1. Mol Neurobiol. 2017;54:2939‐2949. 27023226 10.1007/s12035-016-9874-6

[189] Nadal X , Del Rio C , Casano S , et al. Tetrahydrocannabinolic acid is a potent PPARgamma agonist with neuroprotective activity. Br J Pharmacol. 2017;174:4263‐4276. 28853159 10.1111/bph.14019 PMC5731255

[190] Chen R , Wan J , Song J , Qian Y , Liu Y , Gu S . Rational screening of peroxisome proliferator‐activated receptor‐gamma agonists from natural products: potential therapeutics for heart failure. Pharm Biol. 2017;55:503‐509. 27937122 10.1080/13880209.2016.1255648 PMC6130577

[191] Kim MH , Kim EJ , Choi YY , Hong J , Yang WM . Lycium chinense improves post‐menopausal obesity via regulation of PPAR‐gamma and estrogen receptor‐alpha/beta expressions. Am J Chin Med. 2017;45:269‐282. 28231739 10.1142/S0192415X17500173

[192] Peng SG , Pang YL , Zhu Q , Kang JH , Liu MX , Wang Z . Chlorogenic acid functions as a novel agonist of PPARgamma2 during the differentiation of mouse 3T3‐L1 preadipocytes. Biomed Res Int. 2018;2018:8594767. 30627576 10.1155/2018/8594767 PMC6304673

[193] Matsubara T , Takakura N , Urata M , et al. Geranylgeraniol induces PPARgamma expression and enhances the biological effects of a PPARgamma agonist in adipocyte lineage cells. In Vivo. 2018;32:1339‐1344. 30348686 10.21873/invivo.11384 PMC6365726

[194] Lu Y , Yao J , Gong C , et al. Gentiopicroside ameliorates diabetic peripheral neuropathy by modulating PPAR‐ Gamma/AMPK/ACC signaling pathway. Cell Physiol Biochem. 2018;50:585‐596. 30308492 10.1159/000494174

[195] Balakrishnan BB , Krishnasamy K , Choi KC . Moringa concanensis Nimmo ameliorates hyperglycemia in 3T3‐L1 adipocytes by upregulating PPAR‐gamma, C/EBP‐alpha via Akt signaling pathway and STZ‐induced diabetic rats. Biomed Pharmacother. 2018;103:719‐728. 29680740 10.1016/j.biopha.2018.04.047

[196] Ochiai M , Takeuchi T , Nozaki T , Ishihara KO , Matsuo T . Kaempferia parviflora ethanol extract, a peroxisome proliferator‐activated receptor gamma ligand‐binding agonist, improves glucose tolerance and suppresses fat accumulation in diabetic NSY mice. J Food Sci. 2019;84:339‐348. 30726580 10.1111/1750-3841.14437

[197] Balakrishnan BB , Krishnasamy K , Mayakrishnan V , Selvaraj A . Moringa concanensis Nimmo extracts ameliorates hyperglycemia‐mediated oxidative stress and upregulates PPARgamma and GLUT4 gene expression in liver and pancreas of streptozotocin‐nicotinamide induced diabetic rats. Biomed Pharmacother. 2019;112:108688. 30798121 10.1016/j.biopha.2019.108688

[198] Zou G , Gao Z , Wang J , et al. Deoxyelephantopin inhibits cancer cell proliferation and functions as a selective partial agonist against PPARgamma. Biochem Pharmacol. 2008;75:1381‐1392. 18164690 10.1016/j.bcp.2007.11.021

[199] Lu ZH , Peng JH , Zhang RX , et al. Dihydroartemisinin inhibits colon cancer cell viability by inducing apoptosis through up‐regulation of PPARgamma expression. Saudi J Biol Sci. 2018;25:372‐376. 29472793 10.1016/j.sjbs.2017.02.002 PMC5816000

[200] Au‐Yeung KK , Liu PL , Chan C , Wu WY , Lee SS , Ko JK . Herbal isoprenols induce apoptosis in human colon cancer cells through transcriptional activation of PPARgamma. Cancer Invest. 2008;26:708‐717. 18608213 10.1080/07357900801898656

[201] Liu L , Si N , Ma Y , et al. Hydroxysafflor‐yellow A induces human gastric carcinoma BGC‐823 cell apoptosis by activating peroxisome proliferator‐activated receptor gamma (PPARgamma). Med Sci Monit. 2018;24:803‐811. 29417935 10.12659/MSM.905587 PMC5813879

[202] Qu Q , Qu J , Guo Y , Zhou BT , Zhou HH . Luteolin potentiates the sensitivity of colorectal cancer cell lines to oxaliplatin through the PPARgamma/OCTN2 pathway. Anticancer Drugs. 2014;25:1016‐1027. 25075794 10.1097/CAD.0000000000000125

[203] Yang CM , Lu YL , Chen HY , Hu ML . Lycopene and the LXRalpha agonist T0901317 synergistically inhibit the proliferation of androgen‐independent prostate cancer cells via the PPARgamma‐LXRalpha‐ABCA1 pathway. J Nutr Biochem. 2012;23:1155‐1162. 22137263 10.1016/j.jnutbio.2011.06.009

